# TNF****α**** Mediated IL-6 Secretion Is Regulated by JAK/STAT Pathway but Not by MEK Phosphorylation and AKT Phosphorylation in U266 Multiple Myeloma Cells

**DOI:** 10.1155/2013/580135

**Published:** 2013-09-16

**Authors:** Chansu Lee, Jeong-In Oh, Juwon Park, Jee-Hye Choi, Eun-Kyung Bae, Hyun Jung Lee, Woo June Jung, Dong Soon Lee, Kwang-Sung Ahn, Sung-Soo Yoon

**Affiliations:** ^1^Cancer Research Institute, College of Medicine, Seoul National University, 101 Daehak-ro, Jongro-gu, Seoul 110-799, Republic of Korea; ^2^Clinical Research Institute, Seoul National University Hospital, 101 Daehak-ro, Jongro-gu, Seoul 110-799, Republic of Korea; ^3^Hematology and Medical Oncology, Dongguk University Ilsan Hospital, 27 Dongguk-ro, Ilsandong-gu, Goyang-si, Gyeonggi-do 410-773, Republic of Korea; ^4^Department of Laboratory Medicine, Seoul National University Hospital, 101 Daehak-ro, Jongro-gu, Seoul 110-799, Republic of Korea; ^5^Department of Internal Medicine, Seoul National University Hospital, 101 Daehak-ro, Jongro-gu, Seoul 110-799, Republic of Korea

## Abstract

IL-6 and TNF**α** were significantly increased in the bone marrow aspirate samples of patients with active multiple myeloma (MM) compared to those of normal controls. Furthermore, MM patients with advanced aggressive disease had significantly higher levels of IL-6 and TNF**α** than those with MM in plateau phase. TNF**α** increased interleukin-6 (IL-6) production from MM cells. However, the detailed mechanisms involved in signaling pathways by which TNF**α** promotes IL-6 secretion from MM cells are largely unknown. In our study, we found that TNF**α** treatments induce MEK and AKT phosphorylation. TNF**α**-stimulated IL-6 production was abolished by inhibition of JAK2 and IKK**β** or by small interfering RNA (siRNA) targeting TNF receptors (TNFR) but not by MEK, p38, and PI3K inhibitors. Also, TNF**α** increased phosphorylation of STAT3 (ser727) including c-Myc and cyclin D1. Three different types of JAK inhibitors decreased the activation of the previously mentioned pathways. In conclusion, blockage of JAK/STAT-mediated NF-**κ**B activation was highly effective in controlling the growth of MM cells and, consequently, an inhibitor of TNF**α**-mediated IL-6 secretion would be a potential new therapeutic agent for patients with multiple myeloma.

## 1. Introduction

Despite intensive investigations during the past decade, multiple myeloma (MM) remains an incurable disease. One challenge lies in that the bone marrow (BM) microenvironment and the mutual interaction between multiple myeloma cells and bone marrow stromal cells contribute to modulation of biological behaviors of tumor cells in each patient. Therefore, novel therapeutic approaches targeting both tumor cells and stromal cells are being eagerly sought [1–3].

The bone marrow microenvironment is composed of many cellular and cellular components such as endothelial cells, stromal cells, osteoclasts, osteoblast, immune cells, fat cells, and extracellular matrices. These cells interact by direct adhesion or by secretion of numerous cytokines to support tumor progression and confer drug resistance [4–7]. A number of factors present in BM microenvironment have been dysregulated in patients with MM [8–10]. Cytokines including IL-6, VEGF, IGF-1, TNF*α*, and HGF produced from bone marrow stromal cells (BMSCs) and tumor cells directly and/or indirectly influence MM cells in autocrine and paracrine manners. Also, overexpressed cytokines in BM microenvironment eventually contribute to the acquisition of resistance against anticancer drugs [4]. Therefore, biology of interaction between MM cells and BMSCs needs to be verified to improve treatment outcome of MM.

TNF*α* is one of the factors elevated in multiple myeloma patients. As a 7cytokine, TNF*α* is associated with various physiological and pathological processes such as cell growth, apoptosis, and proliferation. Moreover, TNF*α* is known for promoting osteoclastogenesis and inhibiting osteoblastogenesis. It is also responsible for regulating homeostasis in other diseases such as type I diabetes [11] and inflammatory arthritis [12]. The major mechanism by which TNF*α* mediates progression of multiple myeloma cells is via regulation of nuclear factor kappa B (NF-*κ*B) transcription factor. This molecular signaling regulation is also related to IL-6 secretion by stromal cells and osteoblasts which is a potent growth factor for MM cells [13, 14]. Thus, it is of utmost importance to understand signaling pathways of TNF*α* in relation to IL-6 regulation to develop effective therapeutic strategy against MM.

We found that the levels of TNF*α* and IL-6 were elevated in bone marrow aspirates of multiple myeloma patients. We also analyzed the patterns of correlation between TNF*α* and IL-6 and the mechanisms of TNF*α*-induced IL-6 secretion from multiple myeloma cells. Furthermore, we investigated the biological consequences of IL-6 suppression by inhibition of TNF*α*-mediated intracellular signaling. Importantly, JAK/STAT pathway was directly involved in the signal pathways of TNF*α* in relation to IL-6 and inhibitor of NF-*κ*B, TPCK, was most effective compared to other types of inhibitors. 

## 2. Materials and Methods

### 2.1. Cell Culture and Multiple Myeloma Patient Samples

The human multiple myeloma, U266 cells, was generously provided by Dr. Dongsoon Lee (Seoul National University, College of Medicine, Seoul, Republic of Korea). IM9 was obtained from the Korean Cell Line Bank (Chongro-gu, Seoul, Republic of Korea). These cell lines were maintained in RPMI-1640 medium (Gibco-BRL, Gaithersburg, MD, USA) supplemented with sodium pyruvate, essential vitamins, L-glutamine, penicillin (100 U/mL), streptomycin (100 *μ*g/mL) (Gibco, Grand Island, NY, USA), and 10% heat-inactivated fetal bovine serum, except for U266 cell line (cultured in 15% FBS containing medium). The cells were incubated in a highly humidified atmosphere of 5% CO_2_ and 95% air at 37°C. All experiments were conducted using cells in logarithmic growth phase. Clinical and laboratory parameters of 45 patients with multiple myeloma were shown in Table 1. 11 patients had normal radiographic findings of the skeleton and overall; 4 patients died. 

### 2.2. Antibodies and Reagents

Total and phospho form of mTOR, c-Raf, STAT3 (ser727), phospho-MAPK sampler kit, phospho-AKT (ser473) sampler kit, phospho-MEK1/2 (cell signaling technology, Beverly, MA, USA), c-Myc, cyclin D1, phospho-JNK, JNK, and GAPDH (Santa Cruz, CA, USA) were used as primary antibodies. In addition, horseradish peroxidase-conjugated secondary antibody (Jackson ImmunoResearch laboratories Inc., PA, USA) was used. Bay 11-7082((E)3-[(4-merhylphenyl)sulfonyl]-2-propenenitrile), TPCK (N*α*-Tosyl-Phe Chloromethyl Ketone), PDTC (10 Pyrrolidinecarbodithioic Acid, Ammonium Salt), used as NF-*κ*B inhibitors, PI3K inhibitor (LY294002), JNK inhibitor II, MEK1/2 inhibitor (PD98059), p38 MAPK inhibitor (SB203580), JAK inhibitor I/II, and JAK/STAT inhibitor (AG490) were purchased from Calbiochem corp. (San Diego, CA, USA). These inhibitors were dissolved in DMSO as stock solutions, stored at −20°C, and subsequently diluted with serum-free RPMI1640 medium prior to use. Concentrations of various pharmacologic inhibitors were adapted from IC50 values from the manufacturer's manual. Since the treatment duration was just 1 h instead of several days for usual cytotoxic assay, cells were not affected for their viability. Recombinant human TNF*α* was purchased from R&D systems (Minneapolis, MN, USA), rehydrated in phosphate-buffered saline (PBS) containing 0.1% bovine serum albumin, and stored as a stock solution at −20°C.

### 2.3. Western Blot Analysis

Cells stimulated with specific factors and treated for indicated periods were collected and washed using cold phosphate buffered saline (PBS). Cell pellets were lysed in Kinexus protein lysis buffer (containing 20 mM MOPS (pH 7.0), 2 mM EGTA, 5 mM EDTA, 30 mM sodium fluoride, 60 mM *β*-glycerophosphate (pH 7.2), 20 mM sodium pyrophosphate, 1 mM sodium, orthovanadate, 1% Triton X-100, 1 mM PMSF, aprotinin, leupeptin and pepstatin 1 *μ*g/mL), stored in −20°C. Prepared protein samples were separated using 8% sodium dodecyl sulfate-polyacrylamide gel electrophoresis (SDS-PAGE) and electrotransferred onto nitrocellulose membranes (Millipore, Bedford, MA, USA). The membrane was blocked with Tris-buffered saline solution containing 5% Tween-20 (TBS/T) and 5% nonfat dry milk for 1 h in room temperature. Blots on membranes were probed with specific primary antibodies for overnight and diluted secondary antibody (rabbit for 1 : 20,000, mouse for 1 : 10,000) was used for detecting primary antibody. It was enhanced by chemiluminescence reagents (Amersham Pharmacia Biotech, Piscataway, NJ, USA). 

### 2.4. ELISA

IL-2, IL-4, IL-6, IL-10, IL-17, TNF*α*, TGF*β*_1, and IFN*γ* released from multiple myeloma cells were measured using ELISA kit (R&D systems, Minneapolis, MN, USA). Cells were pretreated with specific reagents for indicated period and then cell-free supernatants were harvested and stored in −70°C. Bone marrow aspirates obtained from 45 patients with multiple myeloma were measured for cytokines concentration in accordance with the manufacturer's instructions. The optical density of the samples was determined using a microplate reader set at 450 nm. 

### 2.5. Transfection of TNFR siRNA

Small interference RNA (siRNA) for siGENOME Human TNFR siRNA (M-005197-00) and siGENOME Nontargeting siRNA Pool (D-001206-13) were purchased from Dharmacon (Lafayette, CO.). Transient transfection of U266 was performed using the Human Cell Line Nucleofector Kit C (VACA-1004; Amaxa Biosystems, Gaithersburg, MD), according to the manufacturer's protocols. Briefly, siRNA (5 *μ*g) was added to 1 × 10^6^ U266 cells suspended in 100 *μ*L of Nucleofector TM solution. The mixture was then transferred into the electroporation cuvette and subjected to electroporation using the X-005 program, according to the manufacturer's instructions. Immediately after electroporation, the cells were suspended in 500 *μ*L of cell culture medium and transferred into culture dishes or plates. The transfected cells were then grown until the knocked-down effect of TNFR siRNA was evident by RT-PCR.

### 2.6. Statistical Analysis

The statistical significance of differences observed in experimental arm versus control was analyzed by Student's *t*-test, using the statistical software GraphPad Prism 4 (GraphPad Software, Inc., La Jolla, CA, USA). Statistical significance was set at a level of *P* < 0.05.

## 3. Results

### 3.1. The Cytokine Patterns in the Bone Marrow Environment of Multiple Myeloma Patients

**Figure 1:**
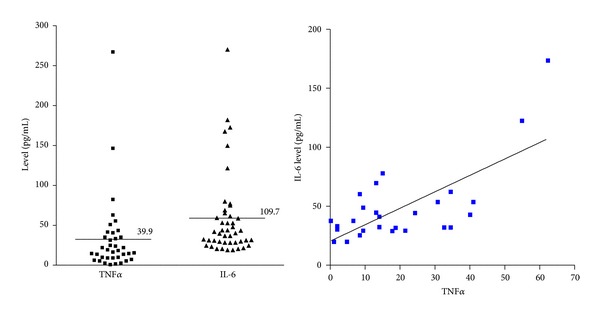
The cytokine patterns in the bone marrow environment of multiple myeloma patients. TNF*α* and IL-6 levels were measured using 45 aspirates of patients with multiple myeloma using ELISA kit. 39.9 pg/mL and 109.7 pg/mL are the mean levels of TNF*α* and IL-6, respectively. The correlation between TNF*α* and IL-6 is significant; *r* ^2^ = 0.4884, *P* < 0.0001.

To identify which among various cytokines are retained with high concentration in the serum of multiple myeloma patients, 8 cytokine (IL-2, IL-4, IL-6, IL-10, IL-17, TNF*α*, TGF*β*_1, and IFN*γ*) levels were measured using 45 bone marrow aspirate samples of multiple myeloma patients (Table 1). Levels of TNF*α*, IL-6, and TGF*β*_1 were elevated, but cytokines such as IL-2, IL-4, and IL-10 were too low to be detected by ELISA. The level of IL-6 showed positive correlation with the level of TNF*α* (Figure 1), and these cytokines showed further correlation with poor prognostic factors such as high level of serum *κ*/*λ* light chain ratio and *β*_2_MG, as well as short overall survival (data not shown).

### 3.2. Effect of TNF*α* on IL-6 Release from Multiple Myeloma

**Figure 2:**
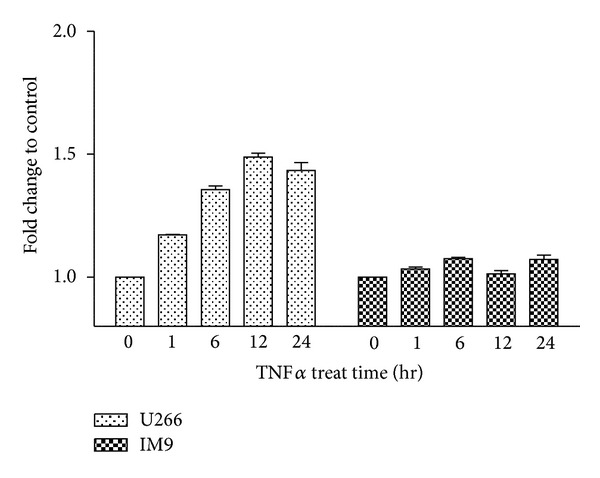
Regulation of IL-6 release by TNF*α* in vitro. After serum starvation, U266 and IM9 multiple myeloma cells were treated with or without 1 ng/mL TNF*α* for indicated times. Cell supernatants from each experimental sample were harvested for ELISA and IL-6 concentrations were determined at different time points with TNF*α* treatment. Relative fold changes compared to TNF*α* nontreated samples as a control were shown in graph. Bars represent the mean ± SEM from three independent experiments.

It has been previously reported that TNF*α* plays a key role in facilitation of IL-6 secretion [15]. Figure 1 showed that there was correlation between IL-6 and TNF*α* level in bone marrow aspirate samples of patients. We examined whether TNF*α* could be the stimulator that releases IL-6 from multiple myeloma cells. In U266 cells, IL-6 secretion was markedly increased in response to TNF*α*, whereas other MM cell lines did not show drastic IL-6 secretion. As shown in Figure 2, IL-6 concentration was 1.5 folds higher in TNF*α*-treated U266 cells compared with untreated cells. However, induction of IL-6 by TNF*α* treatment was not detected in IM9 cells.

### 3.3. Activation of Various Signaling Pathways by TNF*α* and Suppression of IL-6 Release with Inhibitors

**Figure 3:**
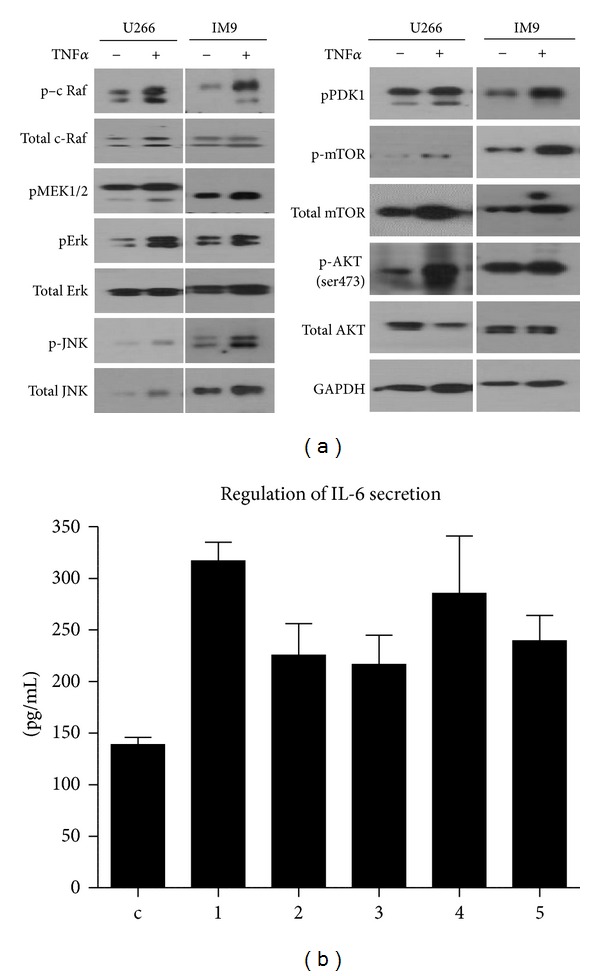
Effect of TNF*α* on activation of MAPK, JNK, and PI3K/AKT signaling pathways and effect of indicated inhibitors on IL-6 secretion. (a) U266 and IM9, multiple myeloma cell lines were starved for 8 h and then treated with or without 1 ng/mL TNF*α* for 10 min. Each cell line was harvested and prepared for immunoblotting. Upregulation of MAPK, JNK, and PI3K/AKT by TNF*α* was examined with indicated antibodies. GAPDH was used to ensure equal loading. (b) U266 cells were pretreated with various signal inhibitors for 1 h, respectively. These cells were then stimulated with TNF*α* for 10 min, and cell supernatants were harvested for ELISA to determine IL-6 concentration induced by TNF*α*. Bars represent the mean ± SEM from three independent experiments. c: control; 1: TNF*α* only; 2: TNF*α* plus 10 *μ*M PD98059; 3: TNF*α* plus 20 *μ*M LY294002; 4: TNF*α* plus 10 *μ*M SB203580; 5: TNF*α* plus 40 nM JNK inhibitor II.

To evaluate the signaling mechanism of TNF*α* on IL-6 secretion, we first examined molecules activated by TNF*α* by western blot analysis in U266 and IM9 cells. As a result of TNF*α* stimulation after serum starvation, various signaling molecules were regulated by TNF*α* including Raf/MEK/Erk, JNK, and PI3K/AKT pathways in both cell lines (Figure 3(a)). Since IM9 showed little difference in the level of IL-6 secretion despite TNF*α* stimulation, we opted to use U266 cell line for further study since the aim of our study was to delineate the role of TNF*α* on the secretion of IL-6. Cells were preincubated with PD98059, LY294002, SB203580, and JNK inhibitor II for suppression of the phosphorylation of p44/42MAPK, PI3K/AKT, p38 MAPK, and JNK, respectively, and then stimulated with TNF*α* for IL-6 release from cells. The inhibitors were not potent in blocking secretion of IL-6 from U266 multiple myeloma cells (Figure 3(b)).

### 3.4. Regulation of IL-6 Secretion Induced by TNF*α* via JAK/STAT Pathway

**Figure 4:**
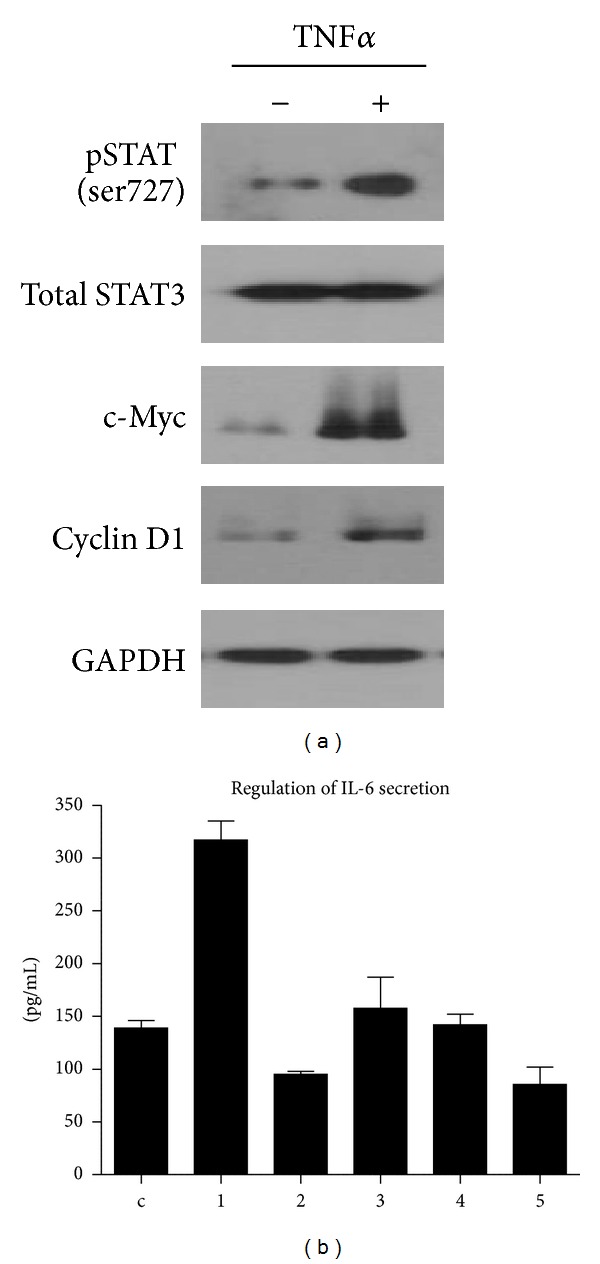
IL-6 secretion by TNF*α* regulated by JAK/STAT pathway. U266 cells were serum starved for 8 h and treated with indicated JAK/STAT inhibitors for 1 h or transfected with TNFR siRNA. The cells were then stimulated with TNF*α* to induce IL-6 secretion and harvested for immunoblotting with indicated antibodies (a). Cell supernatants were collected to determine IL-6 levels using ELISA kit (b). TNF receptor targeted siRNA was used as a positive control. Data shown are the means ± SEM of three independent experiments. c: control; 1: TNF*α* only; 2: TNF*α* plus 20 *μ*M AG490; 3: TNF*α* plus 10 *μ*M JAK inhibitor I; 4: TNF*α* plus 10 *μ*M JAK inhibitor II; 5: TNF*α* plus TNFR siRNA.

To further investigate whether TNF*α* induces the activation of JAK/STAT pathway which is associated with NF-*κ*B activation, TNF*α* mediated STAT3 phosphorylation and the induction of c-myc and cyclin D1, downstream genes of JAK/STAT activation, were examined in U266 cells. As shown in Figure 4(a), TNF*α* induced phospho-STAT3 as well as increased the expression levels of cMyc and cyclin D1. So, we used JAK/STAT inhibitors such as AG490, JAK inhibitor I, and JAK inhibitor II to examine whether IL-6 secretion by TNF*α* is regulated by JAK/STAT pathway. As shown in Figure 4(b), all inhibitors that target JAK/STAT signaling resulted in the suppression of IL-6 secretion by TNF*α*. Similar suppression was also achieved by targeting TNF*α* receptor using siRNA.

### 3.5. Suppression of IL-6 Secretion by TPCK

**Figure 5:**
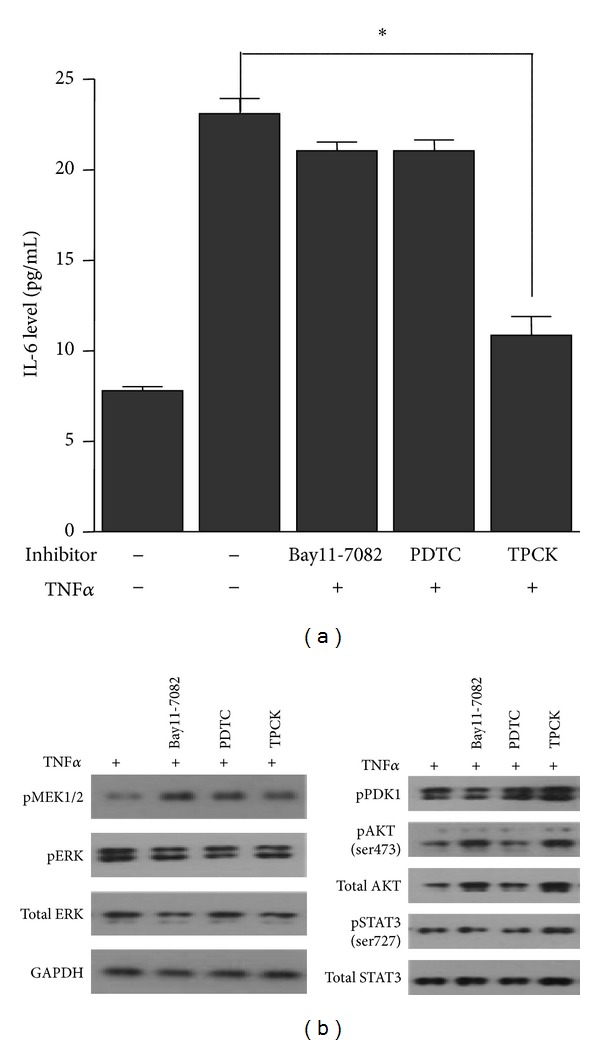
Effect of NF-*κ*B inhibitors on TNF*α* induced IL-6 levels and signaling induction of its upstream pathways. U266 cells were serum starved for 8 hr and pretreated with NF-*κ*B inhibitors (10 *μ*M Bay 11-7082, 10 *μ*M PDTC, 20 *μ*M TPCK) for 1 hr. These cells were then stimulated with 1 ng/mL TNF*α* for 10 min to stimulate IL-6 secretion. Cell supernatants were subjected to determine IL-6 level using ELISA (a), and proteins were prepared for detection of signaling activation using indicated antibodies (b). Data shown are the means ± SEM of three independent experiments.

Numerous studies revealed that TNF*α* induces IL-6 secretion from multiple myeloma cells and bone marrow stromal cells through NF-*κ*B pathway [16]. Moreover, most multiple myeloma cells constitutively activate nuclear NF-*κ*B signaling. So, we examined whether inhibition of NF-*κ*B pathway affects secretion of IL-6 by TNF*α*.  Figure 5(a) describes that only the cells treated with TPCK, which inhibits the DNA binding of transiently expressed p65/RelA by suppression of IKK-beta, effectively decreased the level of IL-6 secretion. To examine whether NF-*κ*B inhibitors directly affect the signaling pathways, such as MEK/ERK, PDK1/AKT, and JAK/STAT, signaling molecules, such as p-ERK, p-PDK1, p-AKT, and p-STAT3, were examined after treating with three different inhibitors.  Figure 5(b) describes that none of the NF-*κ*B inhibitors suppresses TNF*α*-induced phosphorylation of signaling molecules. Since TPCK did not directly inhibit the TNF*α*-induced p-STAT3, we can assume that TPCK action is not mediated by STAT3 pathway but by downstream of JAK/STAT pathway (i.e., NF-*κ*B pathway).

## 4. Discussion

Chemokines and cytokines existed in BM Microenvironment directly and indirectly influence MM cells. The well-known factors including IL-6, TNF*α*, TGF*β*_1, and IFN*γ*, which are produced from BMSCs and MM cells, directly affect the survival and growth of MM cells. Several of proinflammatory factors such as IL-2, IL-4, IL-10, and IL-17 are also reported for their association with survival and growth of MM cells [9, 17, 18].

In this study, we investigated the biological mechanism of action of the TNF*α* in human myeloma cells. TNF*α*, as a proinflammatory cytokine, is associated with various processes for multiple myeloma progression such as cell growth, death, and differentiation [19, 20]. In the aspirates of patients with multiple myeloma, elevated TNF*α* level has been observed in numerous studies and this is correlated with poor prognosis [8, 10]. There are also multiple lines of evidence suggesting that TNF*α* is predictive of progression-free survival after thalidomide therapy in MM patients [21, 22]. Consistent with previous reports, we found that not only TNF*α* but also IL-6 level was highly present in aspirates of patients with multiple myeloma, while no difference was seen with T-cell related or produced cytokines such as IL-2, IL-4, IL-10, IL-17, and IFN *γ* (data not shown). We also verified the correlation between TNF*α* and IL-6.

TNF*α* has a central role in bone pathophysiology along with the receptor activator of NF-*κ*B ligand (RANKL) as a skeletal catabolic agent. It is known to be responsible for promoting osteoclastogenesis and inhibiting osteoblast function. Bone destruction is generated in multiple myeloma patients which is unique from other hematologic malignancies; this phenomenon might be related to increase TNF*α* levels. So we analyzed the correlation patterns between TNF*α* levels and bone lesion development. Patients having higher levels of TNF*α* in bone marrow aspirate developed bone lesions more easily by almost 1.5 folds than those having lower levels of TNF*α* (data not shown), consistent with previous report [23]. In addition to TNF*α*, IL-6, produced from multiple myeloma cells as well as bone marrow stromal cells, is also a major growth factor for tumor cells regulating various biological signaling. One of the mechanisms regulating osteoclastogenesis is through NF-*κ*B-dependent IL-6 secretion. TNF*α* potently stimulates IL-6 secretion by stromal cells and osteoblasts, and accumulated IL-6 stimulates growth of multiple myeloma cells. Furthermore, it mediates the effects of other inflammatory cytokines on osteoclast formation, such as IL-1 and TNF*α* again [16]. Recently, it was shown that it can induce apoptosis of mature osteoblast as well as inhibit the proliferation of mesenchymal stem cells [19]. We found that some of myeloma cells including U266 cells showed stronger response to exogenous IL-6 (data not shown), and these cells also secreted more IL-6 in response to TNF*α* in vitro. So, we hypothesized that TNF*α* is one of the major factors that regulate IL-6 secretion from multiple myeloma cells. First, we examined the signaling pathways that may be the candidate bridge between TNF*α* and IL-6. We found that TNF*α* could regulate cell proliferation, survival, and antiapoptosis by inducing various signaling pathways such as PI3K/AKT, JNK, MAPK, and JAK/STAT pathways. Thus, we blocked above TNF*α*-induced molecules to investigate the major signaling pathway involved in IL-6 secretion. There was no significant change in IL-6 secretion from multiple myeloma cells when PI3K/AKT, JNK, or MAPK pathways were inhibited. However, JAK/STAT inhibitors led to considerable decrease in IL-6 level. It indicated that IL-6 secretion by TNF*α* was mainly dependent on JAK/STAT pathway. Moreover, we demonstrated that TNF*α* up-regulates cyclin D1 as well as c-Myc. These are well known for cell proliferation related to cell cycle progression and antiapoptosis.

NF-*κ*B is known to be correlated with drug-resistant activity [24] and to be a major signaling pathway associated with multiple myeloma pathogenesis. Previous reports showed that not only multiple myeloma cells but also BMSCs adherent to multiple myeloma cells also release IL-6 via NF-*κ*B pathway to support MM cell growth [25]. Therefore, NF-*κ*B activation in BMSCs enhances positive loop with adherent multiple myeloma cells by secretion of growth factors such as IL-6, TNF*α*, and HGF. Therefore, there are numerous studies targeting NF-*κ*B pathway using inhibitors to suppress tumor progression [26–28]. Among the three compounds that inhibit NF-*κ*B pathway, our data showed that only TPCK suppressed IL-6 secretion remarkably from multiple myeloma cells as well as BMSCs. Previous reports showed that TPCK, known as chymotrypsin-like proteases, could be used as an NF-*κ*B pathway inhibitor. It targets serine and cysteine activation loop of IKK beta, resulting in blocking of NF-*κ*B binding to DNA in HeLa cells [29]. In addition, Wang et al. reported that TPCK abolishes constitutive RelA activity and uPA overexpression in pancreatic tumor cell lines with dexamethasone [30]. Although previous report demonstrated that TPCK inhibits TRAIL-mediated caspase activity and PDK/AKT signaling in human prostatic carcinoma cell lines [31], our data indicated that induced AKT signaling by TNF*α* was not affected by TPCK in myeloma cells (Figure 5). These observations do not exclude the possibility that TPCK inhibits other kinases but support the potential of TPCK as an NF-*κ*B inhibitor, especially regulating IL-6 secretion in multiple myeloma cells.

Taken together, we analyzed the correlation of high levels of TNF*α* with several prognostic factors in multiple myeloma patients and the signaling regulation in vitro. In addition, the IL-6 secretion is effectively suppressed by specific inhibitors. TNF*α* and IL-6, as pivotal factors for myeloma, can be ideal targets for therapeutic purpose, either directly or by inhibiting interaction with BMSCs. 

## Figures and Tables

**Table 1:** Median values of the measured parameters in the whole multiple myeloma patients.

Parameters	
Male/female (number)	22/23 (45)
TNF alpha level (pg/mL)	40* (3.17–308.7)^†^
IL-6 level (pg/mL)	109.7 (10.73–2695.7)
Age (yr)	63.3 (33–91)
OS (month)	22 (0.2–71.7)
PLT (10^3^/*μ*L)	231.1 (115–518)
Hb (g/dL)	11.16 (7.7–32.1)
WBC (×10^9^/L)	6780.79 (1190–31530)
Serum *κ*/*λ*	21.72 (0.01–414.39)
Serum Cr	2.35 (0.5–15.2)
Serum Ca	9.39 (7.2–13.3)
LDH (IU/L)	200 (107–392)
*β*_2_MG (serum)	9.48 (1.42–72.4)
Serum albumin	3.32 (1.8–4.8)
Bone lesion (%)	44% (20/45)

*Median; ^†^the parenthesis means the range. OS: overall survival. Hb: Hemoglobin. WBC: white blood cells, *β*_2_MG, beta-2 microglobulin.

## References

[1] Hideshima T, Anderson KC (2002). Molecular mechanisms of novel therapeutic approaches for multiple myeloma. *Nature Reviews Cancer*.

[2] Singhal S, Mehta J (2003). Novel therapies in multiple myeloma. *International Journal of Hematology*.

[3] Hideshima T, Chauhan D, Podar K, Schlossman RL, Richardson P, Anderson KC (2001). Novel therapies targeting the myeloma cell and its bone marrow microenvironment. *Seminars in Oncology*.

[4] Meads MB, Hazlehurst LA, Dalton WS (2008). The bone marrow microenvironment as a tumor sanctuary and contributor to drug resistance. *Clinical Cancer Research*.

[5] Nefedova Y, Landowski TH, Dalton WS (2003). Bone marrow stromal-derived soluble factors and direct cell contact contribute to de novo drug resistance of myeloma cells by distinct mechanisms. *Leukemia*.

[6] Podar K, Richardson PG, Hideshima T, Chauhan D, Anderson KC (2007). The malignant clone and the bone-marrow environment. *Best Practice and Research in Clinical Haematology*.

[7] Mitsiades CS, McMillin DW, Klippel S (2007). The role of the bone marrow microenvironment in the pathophysiology of myeloma and its significance in the development of more effective therapies. *Hematology/Oncology Clinics of North America*.

[8] Alexandrakis MG, Passam FH, Sfiridaki A, Kandidaki E, Roussou P, Kyriakou DS (2003). Elevated serum concentration of hepatocyte growth factor in patients with multiple myeloma: correlation with markers of disease activity. *The American Journal of Hematology*.

[9] Cao Y, Luetkens T, Kobold S (2010). The cytokine/chemokine pattern in the bone marrow environment of multiple myeloma patients. *Experimental Hematology*.

[10] Aggarwal R, Ghobrial IM, Roodman GD (2006). Chemokines in multiple myeloma. *Experimental Hematology*.

[11] Coe LM, Irwin R, Lippner D, McCabe LR (2011). The bone marrow microenvironment contributes to type I diabetes induced osteoblast death. *Journal of Cellular Physiology*.

[12] Lu X, Beck GR, Gilbert LC (2011). Identification of the homeobox protein Prx1 (MHox, Prrx-1) as a regulator of osterix expression and mediator of tumor necrosis factor *α* action in osteoblast differentiation. *Journal of Bone and Mineral Research*.

[13] Bommert K, Bargou RC, Stühmer T (2006). Signalling and survival pathways in multiple myeloma. *European Journal of Cancer*.

[14] Kawano MM, Ishikawa H, Tsuyama N (2002). Growth mechanism of human myeloma cells by interleukin-6. *International Journal of Hematology*.

[15] Devlin RD, Reddy SV, Savino R, Ciliberto G, Roodman GD (1998). IL-6 mediates the effects of IL-1 or TNF, but not PTHrP or 1,25(OH)2D3, on osteoclast-like cell formation in normal human bone marrow cultures. *Journal of Bone and Mineral Research*.

[16] Baud V, Karin M (2009). Is NF-*κ*B a good target for cancer therapy? Hopes and pitfalls. *Nature Reviews Drug Discovery*.

[17] Zdzisińska B, Bojarska-Junak A, Dmoszyńska A, Kandefer-Szerszeń M (2008). Abnormal cytokine production by bone marrow stromal cells of multiple myeloma patients in response to RPMI8226 myeloma cells. *Archivum Immunologiae et Therapiae Experimentalis*.

[18] Prabhala RH, Pelluru D, Fulciniti M (2010). Elevated IL-17 produced by TH17 cells promotes myeloma cell growth and inhibits immune function in multiple myeloma. *Blood*.

[19] Wajant H, Pfizenmaier K, Scheurich P (2003). Tumor necrosis factor signaling. *Cell Death and Differentiation*.

[20] Chen G, Goeddel DV (2002). TNF-R1 signaling: a beautiful pathway. *Science*.

[21] Thompson MA, Witzig TE, Kumar S (2003). Plasma levels of tumour necrosis factor *α* and interleukin-6 predict progression-free survival following thalidomide therapy in patients with previously untreated multiple myeloma. *The British Journal of Haematology*.

[22] Sati HIA, Greaves M, Apperley JF, Russell RGG, Croucher PI (1999). Expression of interleukin-1*β* and tumour necrosis factor-*α* in plasma cells from patients with multiple myeloma. *The British Journal of Haematology*.

[23] Lichtenstein A, Berenson J, Norman D, Chang MP, Carlile A (1989). Production of cytokines by bone marrow cells obtained from patients with multiple myeloma. *Blood*.

[24] Markovina S, Callander NS, O’Connor SL (2008). Bortezomib-resistant nuclear factor-*κ*B activity in multiple myeloma cells. *Molecular Cancer Research*.

[25] Chauhan D, Uchiyama H, Akbarali Y (1996). Multiple myeloma cell adhesion-induced interleukin-6 expression in bone marrow stromal cells involves activation of NF-*κ*B. *Blood*.

[26] Sanda T, Iida S, Ogura H (2005). Growth inhibition of multiple myeloma cells by a novel I*κ*B kinase inhibitor. *Clinical Cancer Research*.

[27] Dai Y, Pei XY, Rahmani M, Conrad DH, Dent P, Grant S (2004). Interruption of the NF-*κ*B pathway by Bay 11-7082 promotes UCN-01-mediated mitochondrial dysfunction and apoptosis in human multiple myeloma cells. *Blood*.

[28] Hideshima T, Neri P, Tassone P (2006). MLN120B, a novel I*κ*B kinase *β* inhibitor, blocks multiple myeloma cell growth in vitro and in vivo. *Clinical Cancer Research*.

[29] Ha KH, Byun MS, Choi J, Jeong J, Lee KJ, Jue DM (2009). N-tosyl-L-phenylalanine chloromethyl ketone inhibits NF-*κ*B activation by blocking specific cysteine residues of I*κ*B kinase *β* and p65/RelA. *Biochemistry*.

[30] Wang W, Abbruzzese JL, Evans DB, Chiao PJ (1999). Overexpression of urokinase-type plasminogen activator in pancreatic adenocarcinoma is regulated by constitutively activated RelA. *Oncogene*.

[31] Rokhlin OW, Guseva NV, Taghiyev AF, Glover RA, Cohen MB (2004). Multiple effects of N-*α*-tosyl-L-phenylalanyl chloromethyl ketone (TPCK) on apoptotic pathways in human prostatic carcinoma cell lines. *Cancer Biology and Therapy*.

